# Single-digit-micrometer thickness wood speaker

**DOI:** 10.1038/s41467-019-13053-0

**Published:** 2019-11-08

**Authors:** Wentao Gan, Chaoji Chen, Hyun-Tae Kim, Zhiwei Lin, Jiaqi Dai, Zhihua Dong, Zhan Zhou, Weiwei Ping, Shuaiming He, Shaoliang Xiao, Miao Yu, Liangbing Hu

**Affiliations:** 1 Department of Materials Science and Engineering, University of Maryland, College Park, MD 20742 USA; 2 Department of Mechanical Engineering, University of Maryland, College Park, MD 20742 USA

**Keywords:** Engineering, Materials science, Materials for devices

## Abstract

Thin films of several microns in thickness are ubiquitously used in packaging, electronics, and acoustic sensors. Here we demonstrate that natural wood can be directly converted into an ultrathin film with a record-small thickness of less than 10 μm through partial delignification followed by densification. Benefiting from this aligned and laminated structure, the ultrathin wood film exhibits excellent mechanical properties with a high tensile strength of 342 MPa and a Young’s modulus of 43.6 GPa, respectively. The material’s ultrathin thickness and exceptional mechanical strength enable excellent acoustic properties with a 1.83-times higher resonance frequency and a 1.25-times greater displacement amplitude than a commercial polypropylene diaphragm found in an audio speaker. As a proof-of-concept, we directly use the ultrathin wood film as a diaphragm in a real speaker that can output music. The ultrathin wood film with excellent mechanical property and acoustic performance is a promising candidate for next-generation acoustic speakers.

## Abstract (web summary)

Thin films of several microns in thickness are ubiquitously used in packaging, electronics, and acoustic sensors. Here the authors demonstrate an ultrathin wood film with an aligned and laminal structure and acoustic properties which allows application of the film as diaphragm for an audio speaker.

## Introduction

Thin films of several microns or even nanometers in thickness are used in a wide range of applications, including solar cells^1^, food packaging^2^, water treatment^3^, personal electronics^4,5^, and acoustic sensors^6^. Acoustic membranes, for example, are generally very thin (micron-scale) and must be mechanically robust with a high modulus to enable a highly sensitive frequency response and high vibrational amplitude. In the past few decades, tremendous efforts have been dedicated to developing various acoustic thin film materials based on plastic^7^, metal^8^, ceramic^9^, and carbon-based materials^10–12^ for the purpose of enhancing the quality of the sound output. In particular, plastic thin films are used ubiquitously in commercial speakers as they are low cost and easy to process with controllable thickness and high modulus. However, most plastic films are difficult to degrade, creating an enormous impact on the environment^13–15^. Furthermore, metal, ceramic, and carbon-based materials demonstrate higher modulus than plastic films, which improves the frequency response of the acoustic membrane. But these compounds are typically higher cost and require complex manufacturing processes that consume large amounts of energy, limiting scalable applications. Therefore, it is desirable, yet highly challenging to prepare a high-performance and biodegradable acoustic thin film in a green and more cost-effective way.

With the emphasis on environmental protection in recent years, natural cellulose-based materials, such as bagasse^16^, wood fibers^17–20^, chitin^21,22^, cotton^23–25^, bacterial cellulose^26^, and lignocellulose^27,28^, have provided an environmentally friendly and rapid way to synthesize thin films from sustainable materials rather than using limited fossil resources. In addition to the advantages of abundance and renewability, cellulose fibers display extraordinary mechanical properties with a theoretical tensile strength up to 7.5 GPa and a Young’s modulus up to 120 GPa, which are even higher than those of common metals, ceramics, and many composites^29,30^. Most cellulose-based materials are generally prepared from bottom-up approaches that involve first breaking down the cellulose fibers by mechanical^31^, chemical^32^, or biological^33^ methods, followed by reconstruction into thin films via filtration, freeze-drying, stretching, or slurry casting. Although the advantages of these bottom-up methods include the ability to control the fiber length and the film structure, the multiple-step manufacturing process consumes large amounts of water, reagents, energy, and time, hindering practical applications. Additionally, it is difficult to maintain the excellent mechanical properties of the elementary cellulose fibers in bottom-up assembled cellulose-based films due to the resulting disordered arrangement of the fibers^34–38^.

**Fig. 1:**
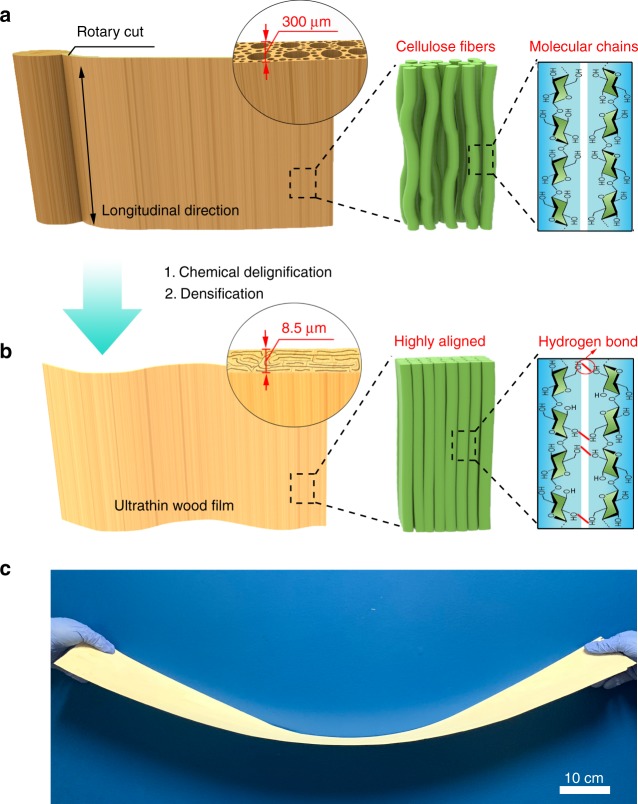
Schematic of the top-down approach to directly transform natural balsa into ultrathin wood films. **a** Left: schematic of the natural wood with its porous structure. Middle: the microstructure of the cellulose fibers in the wood cell walls. Right: the molecular chains of the cellulose fibers. **b** Left: schematic of the ultrathin wood with intertwined, compressed wood channels. Middle: the microstructure of the highly oriented cellulose fibers in the ultrathin wood cell walls. Right: hydrogen bond formation between the neighboring cellulose molecular chains. **c** Meter-long natural balsa wood with a thickness of 300 μm made in the lab

The natural structure of wood provides an effective alternative for more scalable and mechanically robust cellulose films. Featuring aligned cellulose fibers embedded in a soft hydrogel matrix of lignin and hemicellulose within a porous channel structure that runs along the longitudinal direction (Fig. 1a)^39–45^, wood can be utilized as a scaffold for constructing cellulosic thin films via top-down approaches that are more scalable and cost-effective compared to bottom-up methods. The cellulose fibers biosynthesized in the wood cell walls dominate the mechanical properties of wood, while lignin and hemicellulose act as reinforcement agents that tightly bind the cellulose fibers together. Maintaining the alignment of the cellulose fibers rather than breaking them down enables robust mechanical properties of the resulting material. Furthermore, as the most abundant biomass on Earth, wood is renewable and biodegradable, making it more environmentally sustainable than plastics or metals. However, thin cellulosic films made from wood generally have a large thickness of more than 80 µm^27,46^ far from the minimum thickness required for high-performance acoustic application.

Here, we report a facile and large-scale top-down approach for fabricating an ultrathin wood film with a thickness as low as 8.5 µm using a partial delignification and densification process (Fig. 1a). Our approach to synthesizing this membrane involves the partial removal of lignin and hemicellulose from natural balsa wood to generate a higher porosity material while retaining most of the cellulose in the cell walls, which then allows us to densify the treated wood by hot-pressing for a thickness reduction of ~97% (from 300 µm to 8.5 µm, Fig. 1b, Supplementary Fig. 1). Meanwhile, the highly aligned cellulose fibers are densified, which greatly enhances the hydrogen bond formation between the neighboring cellulose molecular chains. The densely packed wood cell wall structure and the highly aligned cellulose fibers contribute to a superior tensile strength and high Young’s modulus. Furthermore, using industry-adopted cutting methods, we are able to fabricate a meter-long natural balsa wood film in the lab (Fig. 1c), revealing this material’s potential for large-scale production upon application of this top-down approach.

## Results

### Morphological and chemical characterizations of wood films

**Fig. 2:**
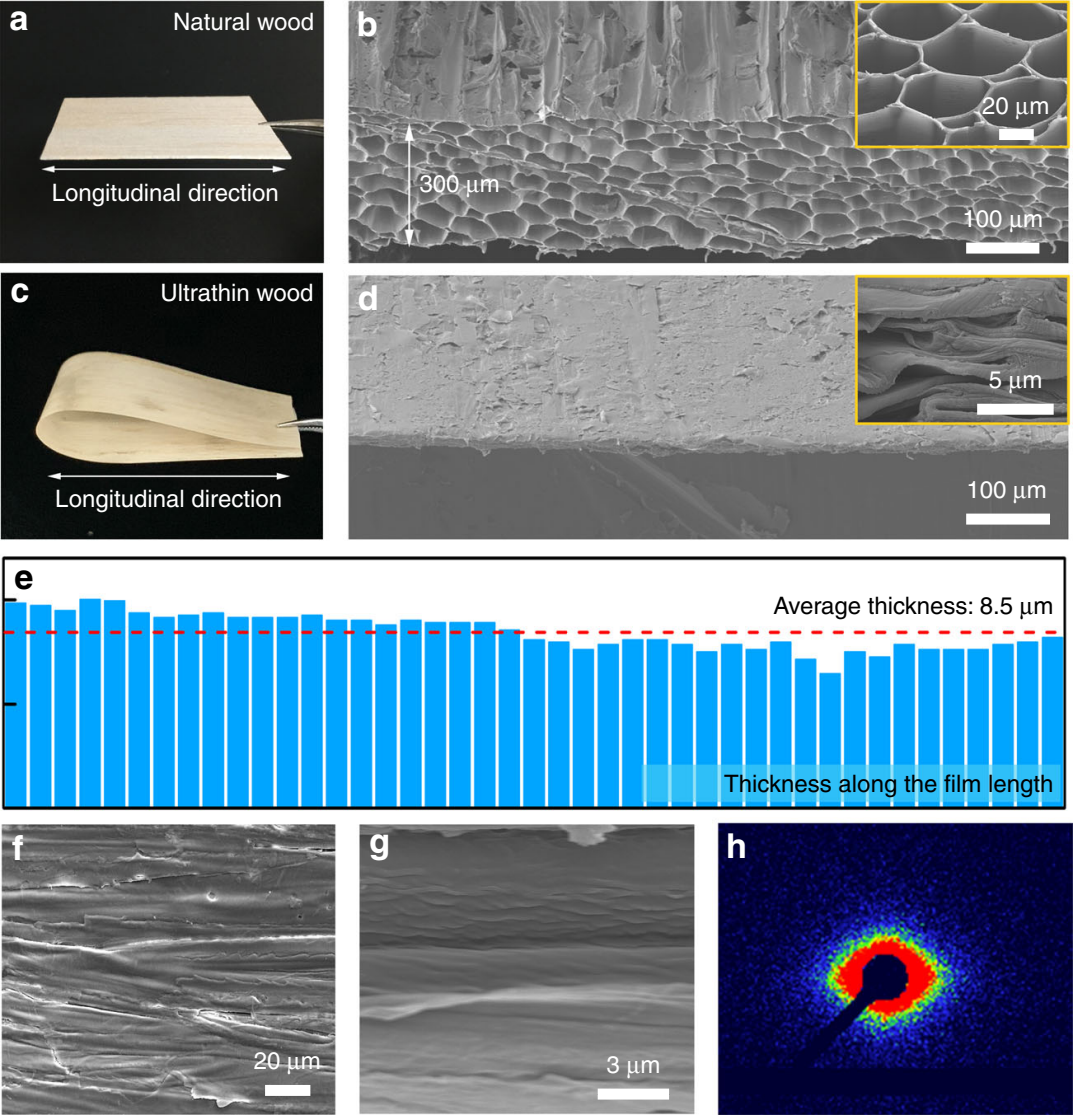
Morphological characterization of wood films. **a** Photograph of the rotary cut natural wood. **b** SEM image of the natural wood, with a thickness of 300 μm. Inset: top-view SEM image of the natural wood, showing its porous wood structure. **c** Photograph of the ultrathin wood. **d** SEM image of the ultrathin wood film, demonstrating its densified wood structure. Inset: Top-view SEM image of the ultrathin wood, revealing its collapsed wood cell walls. **e** The measured thickness of the ultrathin wood along its length at intervals of 5 μm, indicating uniform film thickness. **f**, **g** SEM images of the ultrathin wood, showing the aligned cellulose fibers. **h** Small-angle XRD pattern of the ultrathin wood, indicating the anisotropic alignment of the cellulose nanofibers

Cutting the natural wood along its longitudinal direction maintains the channel structure in the plane of the wood film (Fig. 2a). As shown in Fig. 2b, the microscopic wood channels with irregular polygonal shapes are well arranged along the longitudinal direction. A cross-sectional scanning electron microscopy (SEM) image shows that the thickness of the natural wood is 300 μm (Supplementary Fig. 2). After chemical delignification in a solution containing NaOH and Na_2_SO_3_ (see the Experimental Section for more details), the functional groups assigned to hemicellulose (-(C=O)-) and lignin (-(C=O)-O-) largely decrease compared to the starting material (Supplementary Fig. 3). The component analysis results also show that the lignin and hemicellulose are partially removed by the aqueous solution of NaOH/Na_2_SO_3_ (Supplementary Fig. 4). After partial removal of lignin and hemicellulose from the wood cell walls, the wood channels become soft and a significant amount of cellulose nanofibers are exposed in the cell wall surfaces (Supplementary Fig. 5).

Upon hot-pressing of this delignified material, a flexible and ultrathin wood film was obtained (Fig. 2c), with the porous wood completely converted to a densely packed laminated structure (Fig. 2d). The wood thickness was substantially reduced to 8.5 μm, indicating a reduction of 97% in thickness (Supplementary Fig. 6). Using SEM, we measured the thickness of the ultrathin wood film along its length at intervals of 5 μm and found it was uniformly below 10 μm, as shown in Fig. 2e. As a control, we measured the thickness of the natural wood without chemical treatment after hot-pressing and found it decreased from 300 μm to 100 μm under the same compression conditions, with many wood channels still present in the compressed wood structure (Supplementary Fig. 7). The sharp contrast in the microstructure and thickness between the compressed delignified wood and compressed natural wood highlights the critical role of the partial delignification treatment prior to densification.

Notably, the cellulose nanofibers in the ultrathin wood film remain highly oriented but are more densely laminated than in the natural wood starting material (Fig. 2f, g). X-ray diffraction (XRD) analysis also demonstrates the aligned cellulose molecular chains in the ultrathin wood film. The characteristic peaks around 2*θ* = 16^o^ and 22.6^o^ are assigned to the diffraction peaks of the (110) and (200) planes of the cellulose crystals, respectively (Supplementary Fig. 8). Meanwhile, the small-angle XRD pattern shows the molecular alignment of the cellulose nanofibers (Fig. 2h). These combined results indicate that the partial delignification and densification treatments do not change the crystal structure or arrangement of cellulose nanofibers in the ultrathin wood film, which is important for the material’s mechanical properties.

### Mechanical properties of wood films

**Fig. 3:**
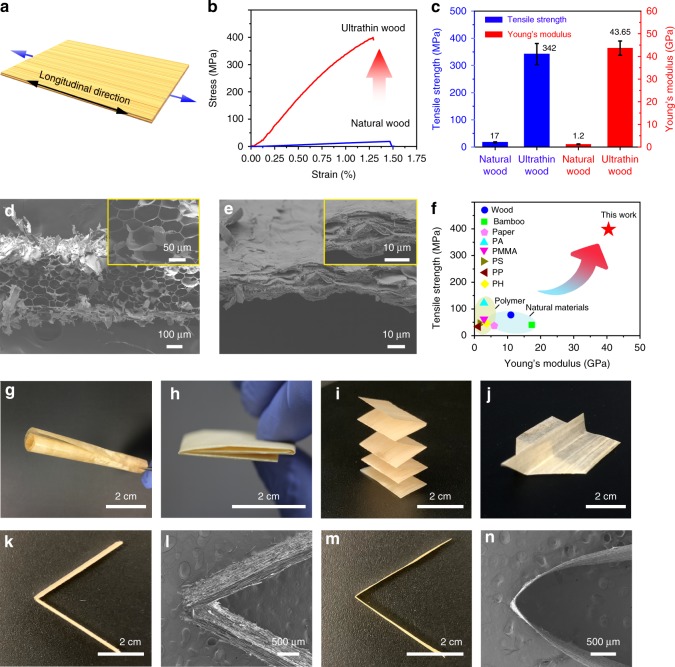
Mechanical properties of wood films. **a** Schematic of the tensile test along the longitudinal direction. **b** Corresponding tensile stress as a function of strain for the natural wood (blue line) and ultrathin wood film (red line). **c** Comparison of the tensile strength and Young’s modulus of the natural wood and ultrathin wood film. Error bars represent standard deviation. **d, e** SEM images of the tensile fracture surface of the natural wood and ultrathin wood film. **f** Comparison of the tensile strength and Young’s modulus of the ultrathin wood film with other widely used polymer and natural materials^50^. (PA: Polyamide; PMMA: Poly (methyl methacrylate); PS: Polystyrene; PP: Polypropylene) **g–j** Photographs of the ultrathin wood film demonstrating its flexibility and various origami designs. **k**, **l** Photograph and SEM image of the natural wood after bending, showing its rigid wood structure. **m**, **n** Photograph and SEM image of the ultrathin wood film after bending, showing its excellent flexibility and folding performance

The resulting ultrathin wood film displays superb mechanical properties that stem from its highly oriented cellulose nanofibers and densely packed microstructure. We conducted mechanical tensile tests to evaluate the material’s mechanical properties (Fig. 3a). Compared with natural wood, the ultrathin wood film shows greatly improved mechanical behavior, with a fracture strength and Young’s modulus (*E*) of up to 342 MPa and 43.65 GPa, respectively (Fig. 3b, c). In contrast, the tensile strength and Young’s modulus of the natural wood are only 17 MPa and 1.2 GPa, respectively. The ultrathin wood film shows almost 20-times improvement in tensile strength and 35-times enhancement in the Young’s modulus. We observed the morphology of the fractured surfaces of the natural wood and ultrathin film using SEM to obtain further insights of the underlying mechanics. In the natural wood slice, the cross-section after the tensile test shows a porous microstructure with numerous wood channels (Fig. 3d). In this material, the cellulose fibers can be easily pulled out from the loosely assembled wood channels under tension, leading to a low fracture strength. In contrast, the wood cell walls in the ultrathin wood film are intertwined together after densification, which not only increases the interfacial area between the wood vessels but also benefits the formation of hydrogen bonds between aligned cellulose nanofibers (Fig. 3e). As a result, compared with the natural wood, the firmly compressed cellulose nanofibers in the ultrathin wood film require more energy to be pulled out. The high tensile strength and Young’s modulus of the ultrathin wood film far exceed that of typical plastic and natural biomaterials, further demonstrating its excellent mechanical properties (Fig. 3f). The ultrathin wood film also possesses great flexibility and foldability, which enables various origami designs (Fig. 3g–j). The ultrathin wood film can be folded into various shapes due to the material’s ultrathin thickness and highly aligned cellulose nanofibers, while the natural wood is brittle and easily broken upon bending and folding (Fig. 3k–n). This folding behavior overcomes the limitations of traditional wood, which is generally unable to support the kind of processing required to form microdevices, suggesting the great potential of using ultrathin wood films for photonics, acoustic sensors, and flexible electronics.

### Acoustic properties and vibration behaviors of wood films

The ultrathin wood film can be an attractive alternative to conventional polymer films for high-performance acoustic transducers. In particular, the high Young’s modulus and ultrathin thickness of the wood film should help to increase the resonance frequency and enhance the displacement amplitude of the diaphragm vibration, respectively^47^. We anticipate that the enhanced vibrational characteristics of our ultrathin wood film will make it highly suitable as a diaphragm for acoustic transducers with a wide operation bandwidth, high sensitivity (for microphones), and high sound pressure level (for speakers)^47–49^.

**Fig. 4:**
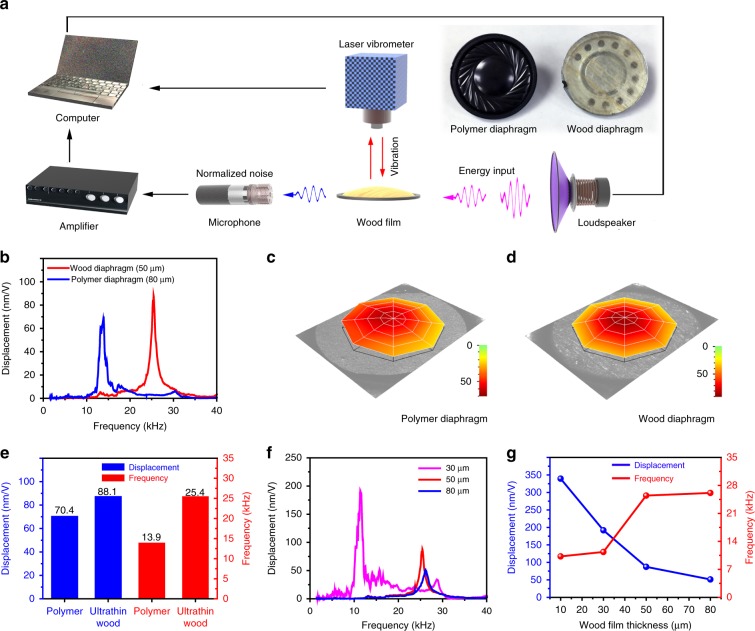
Acoustic properties of wood films. **a** Schematic of the vibrational frequency response measurement system. Insets: photographs of the commercial polymer diaphragm and ultrathin wood film. **b** The vibrational frequency response characteristics of the ultrathin wood film (50 μm) and the commercial polymer (80 μm) diaphragms. **c**, **d** The (0, 1) mode shapes of the polymer (80 μm) and ultrathin wood film (50 μm) diaphragms, respectively. **e** Comparison of the corresponding displacement and first resonance frequency of the ultrathin wood film (50 μm) and polymer (80 μm) diaphragms. **f** The vibrational frequency response characteristics of the ultrathin wood film at different thicknesses. **g** The corresponding displacement and first resonance frequency as a function of the thickness for the wood films

To confirm the increase in the resonance frequency and displacement amplitude of the wood diaphragm, we characterized the frequency response of a vibrating circular diaphragm made of the ultrathin wood film that was 4.3 mm in diameter and compared it with a conventional polymer film (polypropylene) that was obtained from a commercial miniature speaker (Fig. 4a). As shown in Fig. 4b, the wood diaphragm with a thickness of 50 μm has a resonance frequency of 25.4 kHz, which is 1.83-times higher than that of the commercial polymer diaphragm with a thickness of 80 μm (13.9 kHz). It should be noted that the operation frequency bandwidth of diaphragm-based acoustic devices is often limited by its first resonance frequency. The manufactured 50-μm-thick wood diaphragm with a first resonance frequency of 25.4 kHz can cover the entire range of audible frequencies for humans (20 Hz to 20 kHz). These resonances are the first resonance of the diaphragm vibration, which we confirmed by their vibration mode shapes (the (0, 1) vibration mode of a circular diaphragm), as shown in Fig. 4c, d. For the (0,1) vibration mode of a circular diaphragm with a fixed edge, the natural frequency of the diaphragm can be written as^47^

1 $$f_{01} = \frac{{(\alpha _{201}a)^2}}{{2\pi a^2}}\left[ {\frac{{Eh^2}}{{12\rho (1 - \nu ^2)}}} \right]^{1/2}$$

in which *a* and *h* are the radius and thickness of the diaphragm, respectively, *α*_201_*a* is a constant, and *E, ρ*, and *ν* are the Young’s modulus, density, and Poisson’s ratio of the diaphragm material, respectively. From Eq. (1), we can see that the first natural frequency of a circular diaphragm increases with the Young’s modulus and thickness. Although the wood diaphragm is thinner than the polymer diaphragm, its much higher Young’s modulus (more than 10-times higher) results in the observed increase in its natural frequency. On the other hand, the displacement amplitude of a circular diaphragm decreases with the increasing Young’s modulus of the material. The decrease in the displacement amplitude can be compensated by reducing the diaphragm thickness. For example, given the Young’s modulus and damping of a circular diaphragm, a thinner diaphragm renders a larger displacement amplitude, which can be explained by Eqs. ((2)–(4)). Near the first natural frequency, the displacement amplitude at the diaphragm center (*r* = 0) can be expressed as^47^:

2 $$U_0 = \frac{{2\pi pa}}{{\rho hN_1}}\frac{{\left[ {\frac{1}{{\alpha _{201}}}J_1\left( {\alpha _{201}a} \right) - \frac{{J_0\left( {\alpha _{201}a} \right)}}{{\alpha _{101}I_0(\alpha _{101}a)}}I_1\left( {\alpha _{101}a} \right)} \right]\left[ {1 - \frac{{J_0\left( {\alpha _{201}a} \right)}}{{I_0\left( {\alpha _{101}a} \right)}}} \right]}}{{\omega _1\sqrt {\left[ {1 - \left( {\frac{\omega }{{\omega _1}}} \right)^2} \right]^2 \, + \, 4\zeta _1^2\left( {\frac{\omega }{{\omega _1}}} \right)^2} }}$$

3 $$N_1 = {\int \nolimits_0^a} 2\pi r\left[ {J_0\left( {\alpha _{201}r} \right) \, - \, \frac{{J_0\left( {\alpha _{201}a} \right)}}{{I_0\left( {\alpha _{101}a} \right)}}I_0\left( {\alpha _{101}r} \right)} \right]^2dr$$

4 $$\zeta _1 = \frac{\mu }{{\rho h\omega _1}}$$

in which *p* is the pressure amplitude, *ω* is the excitation frequency, *ω*_*1*_ is the first natural frequency, *α*_101_*a* is a constant, and *µ* is the damping coefficient. The displacement amplitude of the 50-μm-thick wood diaphragm near the diaphragm center is 88.1 nm V^−1^ at the resonance frequency, which is 1.25-times higher compared with the conventional polymer diaphragm (70.4 nm V^−1^) (Fig. 4e). Furthermore, we can fabricate various thicknesses of wood films by adjusting the pressure during the hot-pressing process, which allows us to control the resonance frequency and displacement amplitude of the wood diaphragm. Figure 4f shows the frequency responses of 4.3 mm diameter circular wood diaphragms with different thicknesses. The natural wood with a thickness of 300 μm featured no resonance frequency in the range of 0 to 40 kHz (Supplementary Fig. 9). As the diaphragm thickness decreases from 80 μm to 30 μm, the first resonance frequency decreases from 26.2 kHz to 11.5 kHz, and the displacement amplitude at the resonance increases from 47.6 nm V^−1^ to 188.9 nm V^−1^ (Fig. 4f). Further reducing the thickness of the wood film diaphragm to 10 μm results in a displacement amplitude of 340 nm V^−1^ and a first resonance frequency of 10.3 kHz (Supplementary Fig. 10). The corresponding displacement and first frequency of the wood films as function of their thickness is shown in Fig. 4g, indicating that through proper selection of the wood film thickness, a wood diaphragm can provide higher resonance frequency as well as larger displacement amplitude than that of conventional polymer diaphragms, which is highly desirable for high-performance acoustic transducers.

For the tested circular wood diaphragm, although the directional cellulose fibers render the anisotropy in ultrathin wood film, the anisotropy does not affect the (0,1) mode vibration behaviors (Supplementary Fig. 11). The tested wood diaphragm under different direction has an almost identical frequency response regardless its longitudinal direction with respect to the sound wave excitation direction.

### Wood speaker prototype

**Fig. 5:**
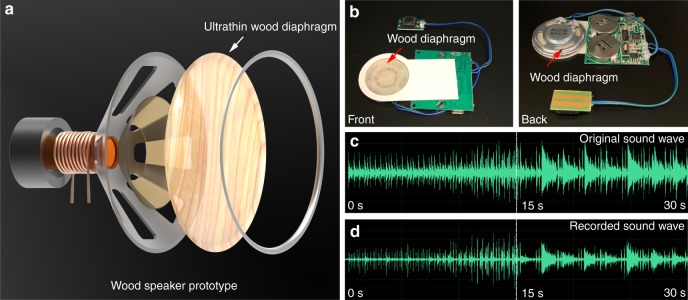
Wood speaker prototype. **a** Schematic of the wood speaker prototype. **b** Photographs of the speaker with the wood diaphragm. **c** The sound wave of the original song (Spain Matador March). **d** The recorded sound wave (Spain Matador March) of the speaker with the wood diaphragm

The diaphragm is a key component in commercial speakers, since their vibration performance determines the quality of the sound source. To demonstrate the promising application of the ultrathin wood film as an acoustic transducer, we assembled a miniature speaker made with the ultrathin wood diaphragm (Fig. 5a). Our prototype was composed of the miniature speaker made with the wood diaphragm (36 mm inner diameter) and a circuit board (Fig. 5b). The miniature speaker also contains a copper coil (14 mm inner diameter) and permanent magnet. We bound the diaphragm and copper coil together and placed them in front of the permanent magnet (Supplementary Fig. 12). When the electric current flows through the copper coil, the direction of its magnetic field will rapidly change, while the permanent magnetic field remains constant. As a result, the electromagnetic forces act on the coil, causing the diaphragm to vibrate back and forth. Thus, the electrical signal is translated into an audible sound through the change in air pressure caused by the diaphragm. The audible sound of the speaker prototype was recorded by a microphone and the sound wave was analyzed through Adobe Audition CC. Compared with the original audio file, the speaker made by the ultrathin wood diaphragm can also play beautiful music (recording of a Spain Matador March, Supplementary Movie 1). Similarly, the sound wave recorded by the microphone from the wood diaphragm shows a similar waveform to that of the original sound wave, suggesting the intriguing application of the ultrathin wood film as a diaphragm in a loud speaker (Fig. 5c, d). Furthermore, this wood speaker may be further improved by the proper diaphragm structure design and the precise assemblies in existing industrial processing. Implementation of this speaker design using our ultrathin wood film may also compete with other technologies in the manufacture of microphones, hearing aids, and acoustic sensors.

## Discussion

We demonstrate an effective top-down strategy for the fabrication of an ultrathin wood film with a thickness of less than 10 μm directly from natural wood (a record-small thickness) via delignification and densification. The ultrathin wood film has a unique microstructure with intertwined wood cell walls and aligned cellulose nanofibers, which contribute to its outstanding mechanical properties with a more than 20-times increase in tensile strength (up to 342 MPa) and 35-times enhancement in Young’s modulus (43.6 GPa). Compared with a commercial polypropylene diaphragm, using the strong and ultrathin wood film we can achieve a high-performance acoustic transducer with 1.83-times increased resonance frequency and 1.25-times enhanced displacement amplitude. We also demonstrate a loud speaker prototype that can generate music by the vibration of the ultrathin wood diaphragm, suggesting this material’s great potential in acoustic applications, such as loud speakers, microphones, and hearing aids. The unique wood structure, ultrathin thickness, and remarkable mechanical properties can potentially enable many other micro designs as well, including wearable energy storage, ion-exchange membranes, food packaging, sensors, and catalyst supports. This demonstrated simple and scalable top-down approach may open up more potential functions and applications of strong film materials from abundant and biodegradable natural resources beyond plastic, metals, and ceramics.

## Methods

### Materials and chemicals

Ultra-light balsa wood sheets with sizes of 10 cm × 10 cm × 0.4 mm were purchased from Specialized BALSA WOOD, LLC. The commercial speaker films were bought from Cylewet 2Pcs Loudspeaker via Amazon. The 120-grit sandpaper was purchased from LECO Corporation. Reagent grade NaOH (Sigma-Aldrich, reagent grade) and Na_2_SO_3_ (Sigma-Aldrich, reagent grade) were used for the partial removal of lignin from the wood.

### Fabrication process of the ultrathin wood film

The natural balsa wood slices with sizes of 10 cm × 10 cm in width and length were polished to 0.3 mm thick using the 120-grit sandpaper. Then the wood slices were immersed and boiled in 2.5 M NaOH and 0.4 M Na_2_SO_3_ solution for 1 h. The processed wood slices were pressed at 100 °C under the pressure between 10 and 15 MPa for 24 h to obtain the ultrathin wood film.

### Mechanical testing

The tensile properties and Young’s Modulus of the wood samples were measured using a 30 kN Instron Testing Machine under ambient conditions. The dimensions for the natural balsa wood and ultrathin wood film samples were 100 mm × 5 mm × 0.4 mm and 100 mm × 5 mm × 0.01 mm, respectively. The samples were clamped and stretched along the longitudinal direction and pulled apart at a constant speed of 5 mm min^−1^.

### Vibration characteristics of the wood films

Circular wood diaphragms were prepared by bonding the wood film to a M4 washer (inner diameter: 4.3 mm, outer diameter: 9.0 mm) using epoxy resin. The vibration of the wood diaphragms was characterized using scanning laser vibrometry. The prepared wood diaphragm was set up under the laser vibrometer (MSA-500, Polytec) and excited by white-noise sound with a 1.5–50 kHz frequency range, which was generated by a speaker (Petterson, LP 400). The frequency responses of the wood diaphragms were obtained by normalizing the vibrometer output against the output of a reference microphone (4191, Bruel & Kjaer) adjacent to the wood diaphragm. All tests were carried out under atmospheric conditions. The room temperature and the relative humidity is 24 °C and 50%, respectively.

### Miniature speaker prototype

The circuit board and miniature speaker were purchased from Shenzhen Maiyout Technologu Co., Ltd (MY-H1650) (Supplementary Fig. 12). The product details are shown in Supplementary Fig. 13. The polypropylene diaphragm (36 mm in diameter) was replaced by the ultrathin wood film with a thickness of 50 μm (36 mm in diameter). Devcon epoxy resin (No. 14250) was used to bond the copper coils and wood diaphragm. The output sound was recorded by an iPhone XS placed at a distance of 10 cm from the miniature speaker. The sound wave was analyzed through Adobe Audition CC.

## Supplementary information

Supplementary Information

Peer Review File

Supplementary Movie 1

## Data Availability

The data that support the findings of this study are available from the corresponding author upon reasonable request.

## References

[1] Yang WS (2017). Iodide management in formamidinium-lead-halide–based perovskite layers for efficient solar cells. Science.

[2] Elvers D, Song CH, Steinbüchel A, Leker J (2016). Technology trends in biodegradable polymers: evidence from patent analysis. Polym. Rev..

[3] Yin J, Deng B (2015). Polymer-matrix nanocomposite membranes for water treatment. J. Membr. Sci..

[4] Lipomi DJ (2011). Skin-like pressure and strain sensors based on transparent elastic films of carbon nanotubes. Nat. Nanotechnol..

[5] Pan XF (2018). Transforming ground mica into high-performance biomimetic polymeric mica film. Nat. Commun..

[6] Mannsfeld SC (2010). Highly sensitive flexible pressure sensors with microstructured rubber dielectric layers. Nat. Mater..

[7] Yang J (2014). Triboelectrification-based organic film nanogenerator for acoustic energy harvesting and self-powered active acoustic sensing. ACS Nano.

[8] Huang CH (2011). Implementation of the CMOS MEMS condenser microphone with corrugated metal diaphragm and silicon back-plate. Sensors.

[9] Yuan L, Zhang Y, Liu J, Xiao H (2017). Ultrafast laser ablation of silica optical fibers for fabrication of diaphragm/cantilever-based acoustic sensors. J. Laser Appl..

[10] Xu SC (2013). Flexible and transparent graphene-based loudspeakers. Appl. Phys. Lett..

[11] Zhou Q, Zettl A (2013). Electrostatic graphene loudspeaker. Appl. Phys. Lett..

[12] Zhou Q (2015). Graphene electrostatic microphone and ultrasonic radio. Proc. Natl Acad. Sci. USA.

[13] Ying L, Huang F, Bazan GC (2017). Regioregular narrow-bandgap-conjugated polymers for plastic electronics. Nat. Commun..

[14] Geyer R, Jambeck JR, Law KL (2017). Production, use, and fate of all plastics ever made. Sci. Adv..

[15] Someya T, Bao Z, Malliaras GG (2016). The rise of plastic bioelectronics. Nature.

[16] Ghaderi M, Mousavi M, Yousefi H, Labbafi M (2014). All-cellulose nanocomposite film made from bagasse cellulose nanofibers for food packaging application. Carbohydr. Polym..

[17] Nogi M, Iwamoto S, Nakagaito AN, Yano H (2009). Optically transparent nanofiber paper. Adv. Mater..

[18] Wang Y, Heim LO, Xu Y, Buntkowsky G, Zhang K (2015). Transparent, stimuli‐responsive films from cellulose‐based organogel nanoparticles. Adv. Funct. Mater..

[19] Tardy Blaise L., Mattos Bruno D., Greca Luiz G., Kämäräinen Tero, Klockars Konrad W., Rojas Orlando J. (2019). Tessellation of Chiral‐Nematic Cellulose Nanocrystal Films by Microtemplating. Advanced Functional Materials.

[20] Reyes G, Borghei M, King AW, Lahti J, Rojas OJ (2018). Solvent welding and imprinting cellulose nanofiber films using ionic liquids. Biomacromolecules.

[21] Mushi NE, Nishino T, Berglund LA, Zhou Q (2018). Strong and tough chitin film from α-chitin nanofibers prepared by high pressure homogenization and chitosan addition. ACS Sustain. Chem. Eng..

[22] Huang J, Zhong Y, Zhang L, Cai J (2017). Extremely strong and transparent chitin films: a high-efficiency, energy-saving, and “green” route using an aqueous KOH/urea solution. Adv. Funct. Mater..

[23] Sadasivuni KK (2015). Transparent and flexible cellulose nanocrystal/reduced graphene oxide film for proximity sensing. Small.

[24] Cheng Q (2018). Construction of transparent cellulose-based nanocomposite papers and potential application in flexible solar cells. ACS Sustain. Chem. Eng..

[25] Ye Dongdong, Lei Xiaojuan, Li Tian, Cheng Qiaoyun, Chang Chunyu, Hu Liangbing, Zhang Lina (2019). Ultrahigh Tough, Super Clear, and Highly Anisotropic Nanofiber-Structured Regenerated Cellulose Films. ACS Nano.

[26] Nogi M, Yano H (2008). Transparent nanocomposites based on cellulose produced by bacteria offer potential innovation in the electronics device industry. Adv. Mater..

[27] Zhu M (2017). Anisotropic, transparent films with aligned cellulose nanofibers. Adv. Mater..

[28] Li Y (2016). Facile extraction of cellulose nanocrystals from wood using ethanol and peroxide solvothermal pretreatment followed by ultrasonic nanofibrillation. Green Chem..

[29] Moon RJ, Martini A, Nairn J, Simonsen J, Youngblood J (2011). Cellulose nanomaterials review: structure, properties and nanocomposites. Chem. Soc. Rev..

[30] Wang S (2017). Super-strong, super-stiff macrofibers with aligned, long bacterial cellulose nanofibers. Adv. Mater..

[31] Jonoobi M (2015). Different preparation methods and properties of nanostructured cellulose from various natural resources and residues: a review. Cellulose.

[32] Chen L, Zhu JY, Baez C, Kitin P, Elder T (2016). Highly thermal-stable and functional cellulose nanocrystals and nanofibrils produced using fully recyclable organic acids. Green Chem..

[33] Sacui IA (2014). Comparison of the properties of cellulose nanocrystals and cellulose nanofibrils isolated from bacteria, tunicate, and wood processed using acid, enzymatic, mechanical, and oxidative methods. ACS Appl. Mater. Inter..

[34] Mittal N (2018). Multiscale control of nanocellulose assembly: Transferring remarkable nanoscale fibril mechanics to macroscale fibers. ACS Nano.

[35] Fu F (2015). Construction of cellulose based ZnO nanocomposite films with antibacterial properties through one-step coagulation. ACS Appl. Mater. Inter..

[36] Qiu C (2018). Super strong all-cellulose composite filaments by combination of inducing nanofiber formation and adding nanofibrillated cellulose. Biomacromolecules.

[37] Yao K, Meng Q, Bulone V, Zhou Q (2017). Flexible and responsive chiral nematic cellulose nanocrystal/poly (ethylene glycol) composite films with uniform and tunable structural color. Adv. Mater..

[38] Morimune-Moriya S, Salajkova M, Zhou Q, Nishino T, Berglund LA (2018). Reinforcement effects from nanodiamond in cellulose nanofibril films. Biomacromolecules.

[39] Song J (2018). Processing bulk natural wood into a high-performance structural material. Nature.

[40] Berglund LA, Burgert I (2018). Bioinspired wood nanotechnology for functional materials. Adv. Mater..

[41] Li T (2018). Anisotropic, lightweight, strong, and super thermally insulating nanowood with naturally aligned nanocellulose. Sci. Adv..

[42] Yu ZL (2018). Bioinspired polymeric woods. Sci. Adv..

[43] Li SC (2018). Wood-derived ultrathin carbon nanofiber aerogels. Angew. Chem. Int. Ed..

[44] Fu Q, Ansari F, Zhou Q, Berglund LA (2018). Wood nanotechnology for strong, mesoporous, and hydrophobic biocomposites for selective separation of oil/water mixtures. ACS nano.

[45] Gan W (2019). Dense, self-formed char layer enables a fire-retardant wood structural material. Adv. Funct. Mater..

[46] Jia C (2017). Scalable, anisotropic transparent paper directly from wood for light management in solar cells. Nano Energy.

[47] Yu M, Balachandran B (2005). Sensor diaphragm under initial tension: linear analysis. Exp. Mech..

[48] Yu M, Balachandran B (2003). Acoustic measurements using a fiber optic sensor system. J. Intell. Mater. Syst. Struct..

[49] Chiang HY, Huang YH (2015). Vibration and sound radiation of an electrostatic speaker based on circular diaphragm. J. Acoust. Soc. Am..

[50] CES. Cambridge Engineering Selector Edupack Software (Granta Design Limited, Cambridge, UK, 2017).

