# Supplementary material for: Single-digit-micrometer thickness wood speaker
Source: Nat Commun. 2019 Nov 8;10:5084. doi: 10.1038/s41467-019-13053-0 (PMC6841728; doi:10.1038/s41467-019-13053-0)
Supplement: Supplementary file 1 — Supplementary Information [file 41467_2019_13053_MOESM1_ESM.pdf]

Supplementary Information

Single-Digit-Micrometer Thickness Wood Speaker

Wentao Gan,[1a] Chaoji Chen,[1a] Hyun-Tae Kim,[2a] Zhiwei Lin,[1] Jiaqi Dai,[1] Zhihua Dong,[1] Zhan Zhou,[1] Weiwei Ping,[1] Shuaiming He,[1] Shaoliang Xiao,[1] Miao Yu,[2,*] Liangbing Hu[1,*]

1. Department of Materials Science and Engineering, University of Maryland, College Park, Maryland, 20742
2. Department of Mechanical Engineering, University of Maryland, College Park, Maryland, 20742

[*] Email: binghu@umd.edu; mmyu@umd.edu

[a] These authors contributed equally to this work.

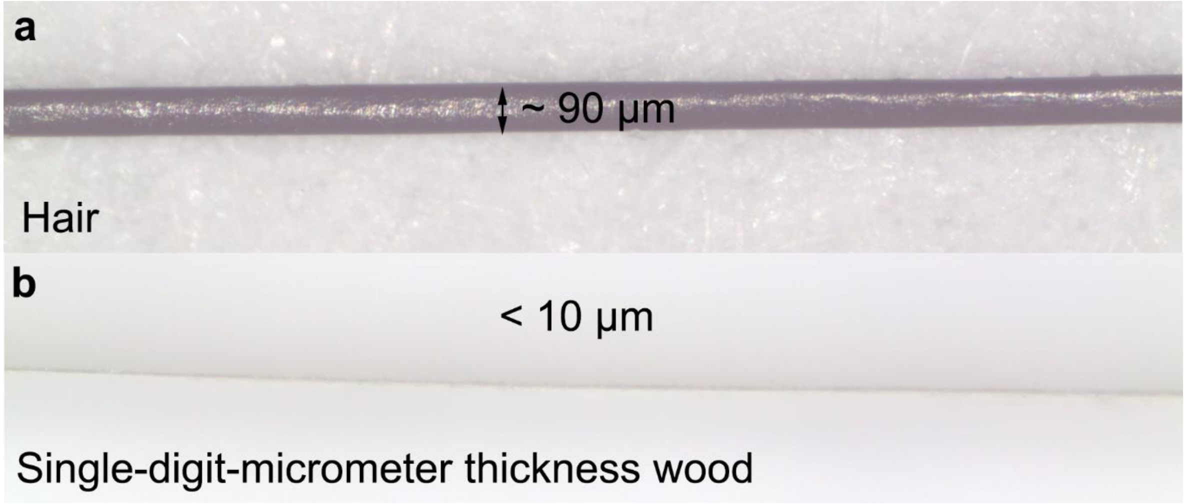

Supplementary Figure 1. (a) Optical micrograph of single hair with a diameter of 90 μm. (b) Optical micrograph of ultrathin wood film with single-digit-micrometer thickness (< 10 μm).

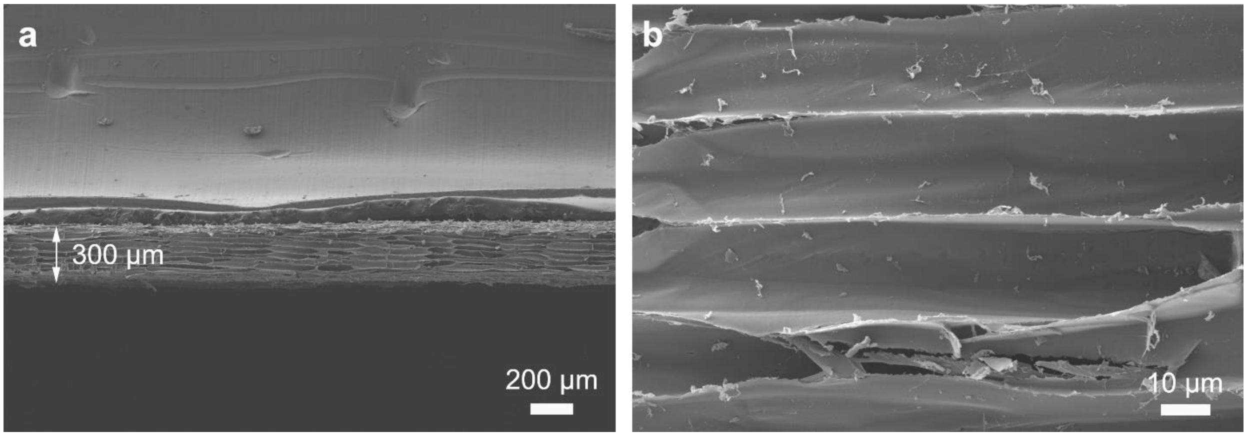

Supplementary Figure 2. (a) Cross-sectional SEM images of natural balsa wood with a thickness of 300 μm. (b) High-magnification cross-sectional SEM image of the natural balsa wood.

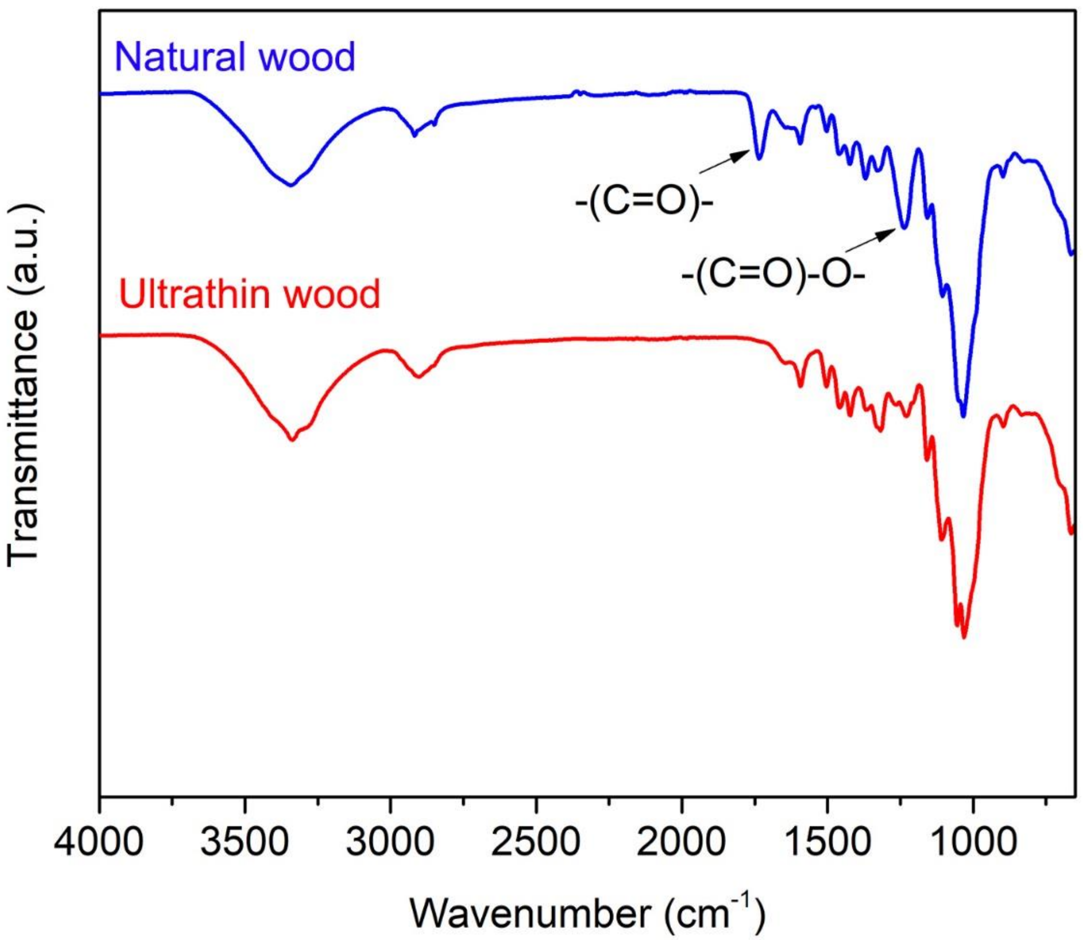

Supplementary Figure 3. FTIR spectra of the natural wood and ultrathin wood. The bands at 1736 and 1245 cm^{-1} of the natural wood are attributed to the characteristic vibration of hemicellulose (-(C=O)-) and lignin (-(C=O)-O-), respectively.

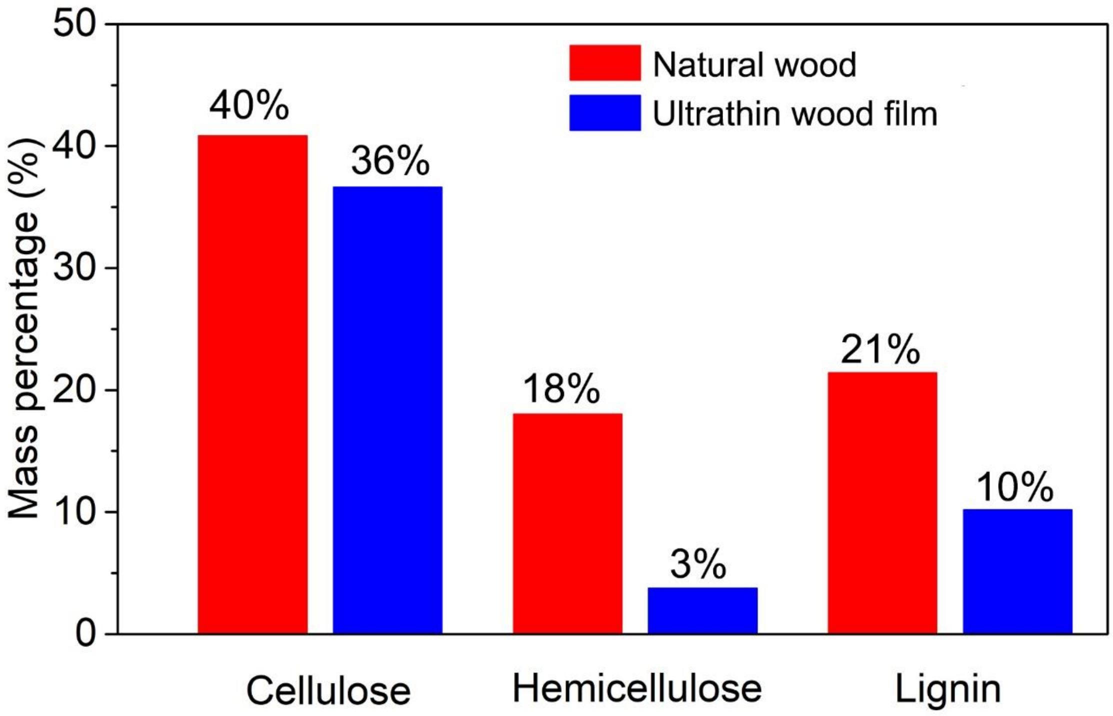

Supplementary Figure 4. The cellulose, hemicellulose, and lignin contents of the natural and ultrathin wood materials, revealing the partial removal of hemicellulose and lignin after chemical treatment.

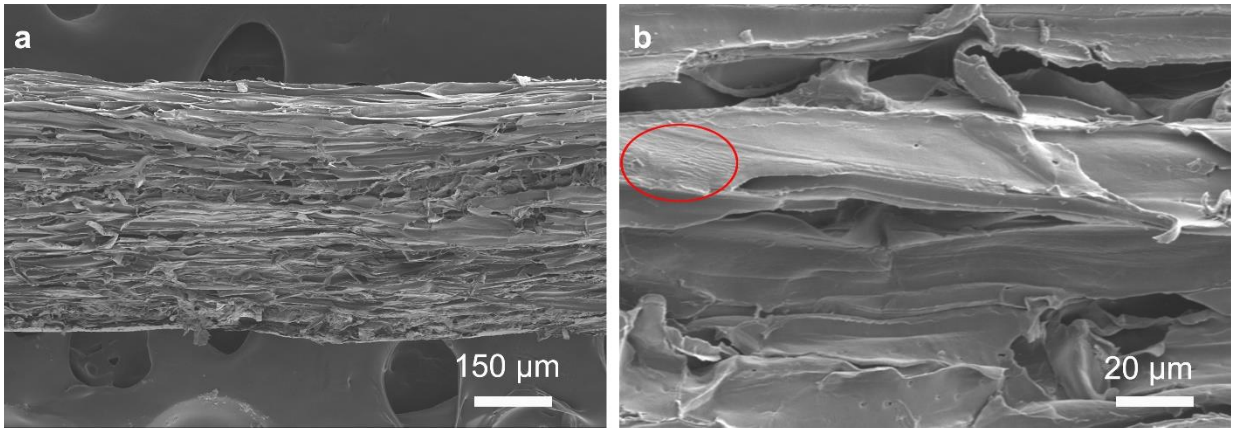

Supplementary Figure 5. (a) Cross-sectional SEM image of the balsa wood after partial delignification, showing the softened channels. (b) High-magnification cross-sectional SEM image of the balsa wood after partial delignification, showing the exposed cellulose fibers (circled area).

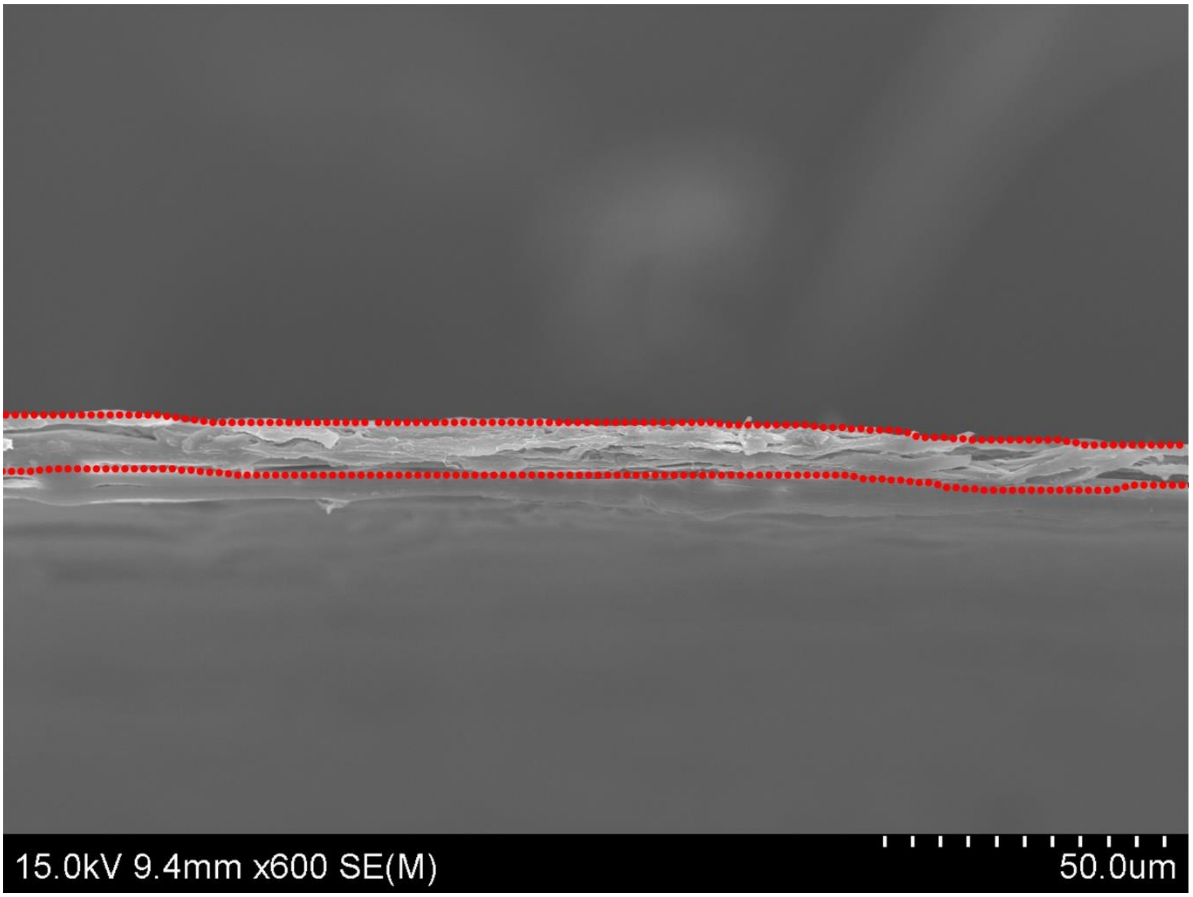

Supplementary Figure 6. Cross-sectional SEM image of the ultrathin wood film with a thickness of 8.5 μm.

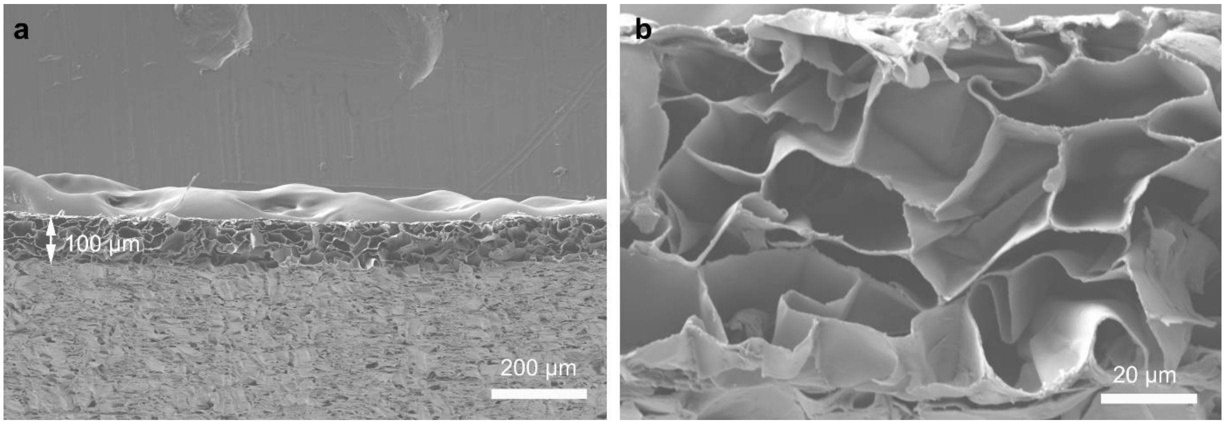

Supplementary Figure 7. SEM images of the natural wood without delignification after hot-pressing, showing its ~100 μm thickness and porous structure.

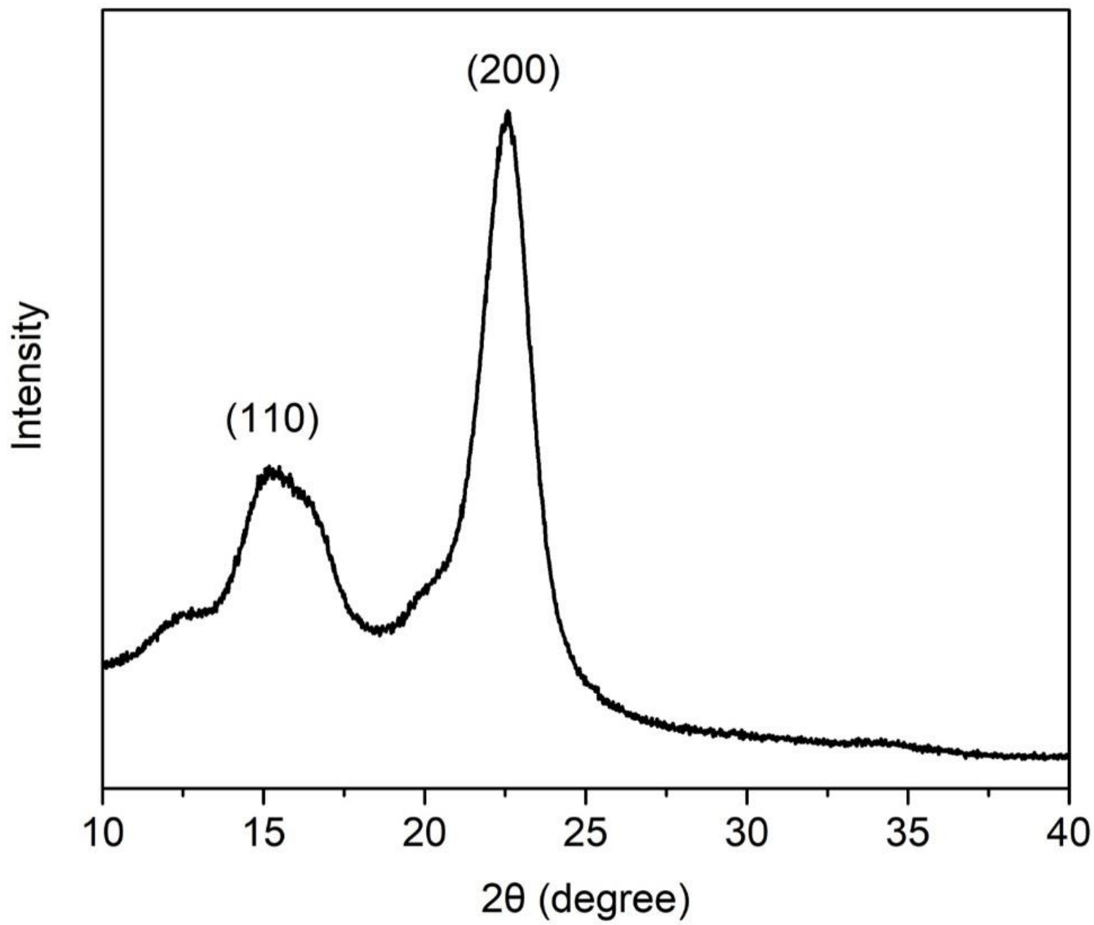

Supplementary Figure 8. XRD of the ultrathin wood. The sharp peaks at 2θ = 16° and 22.6^{o} show the typical crystal structure of cellulose.

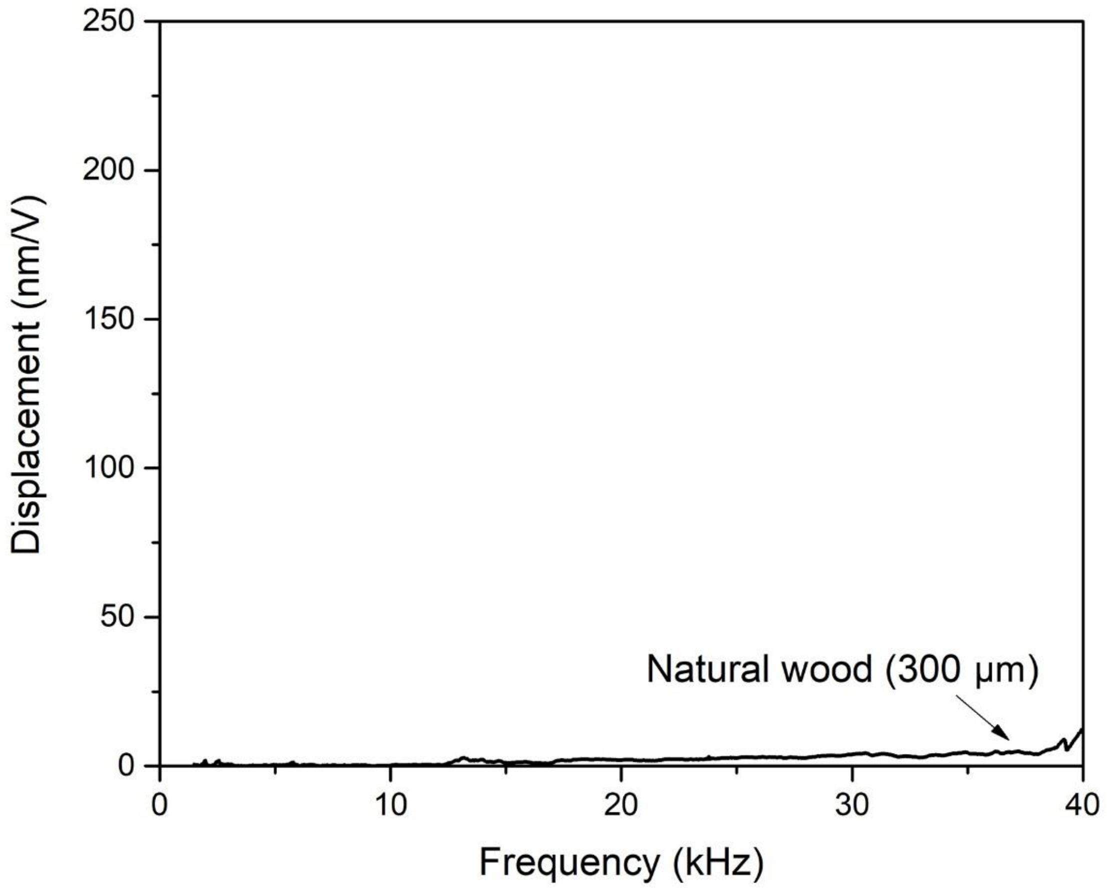

Supplementary Figure 9. The natural wood (300 μm) shows no vibrational frequency response characteristics.

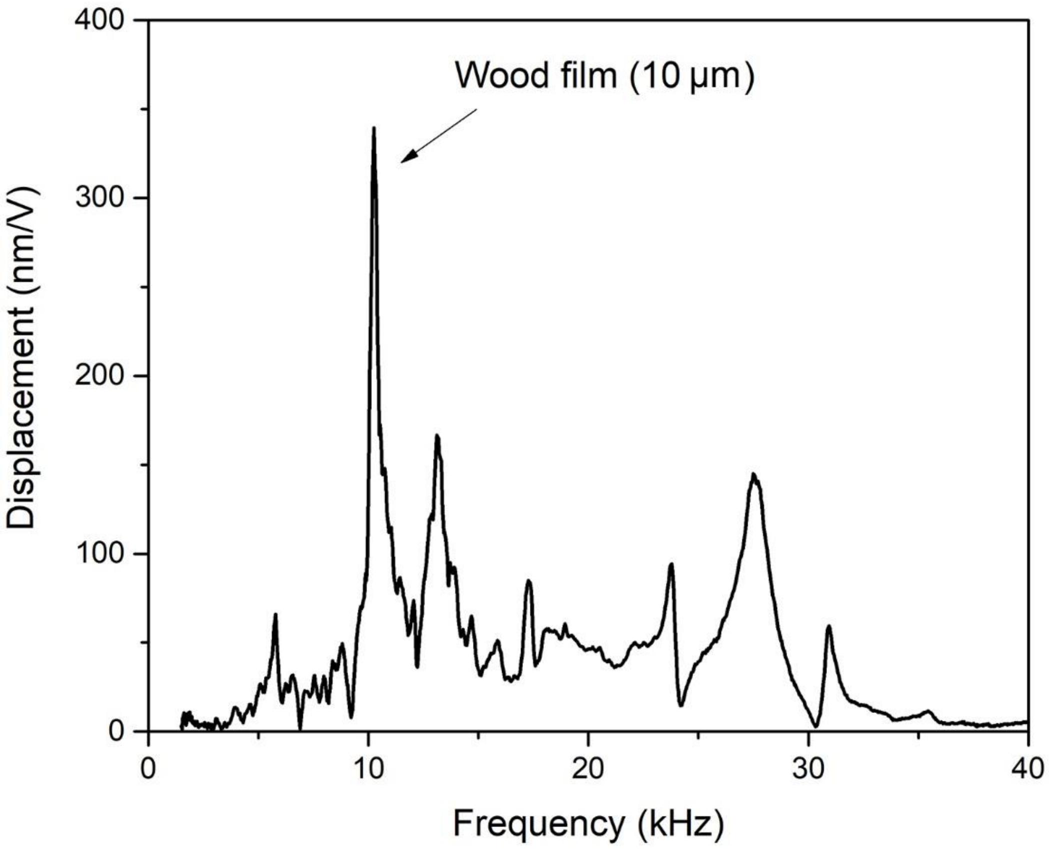

Supplementary Figure 10. The ultrathin wood film (10 μm) shows excellent vibrational frequency response characteristics, with a displacement amplitude of 340 nm V^{-1} and a first resonance frequency of 10.3 kHz. Limited by the instrument accuracy, it is difficult to normalize the noise peaks due to the high sensitivity to frequency vibration.

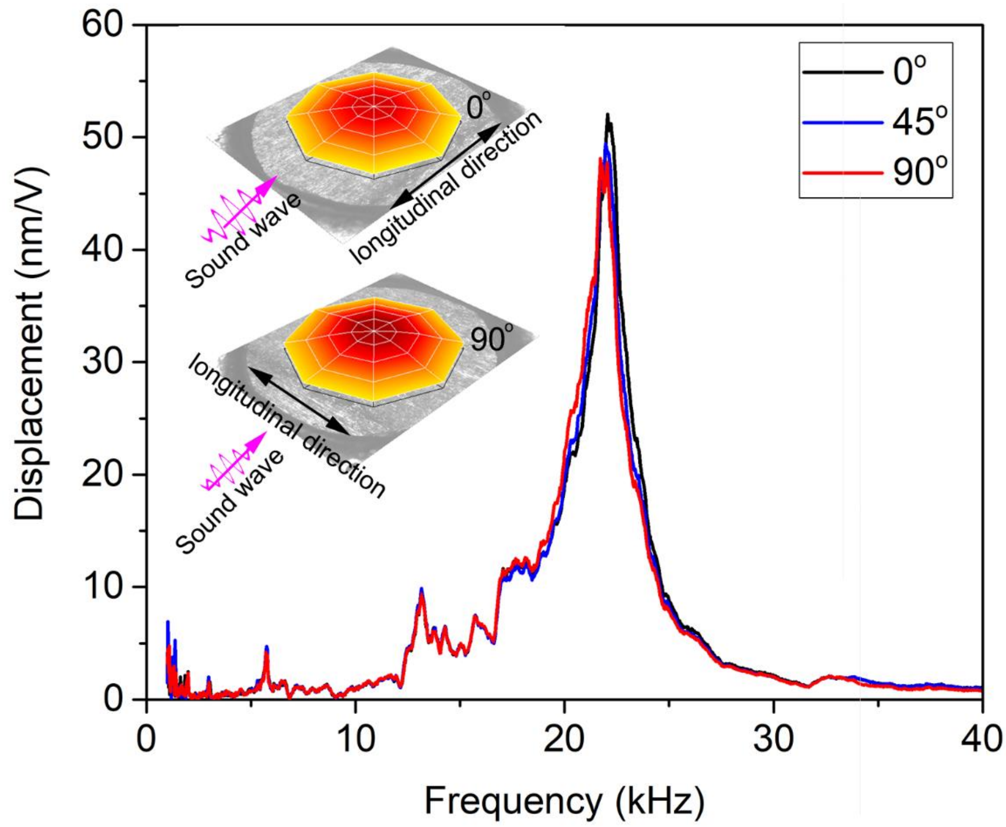

Supplementary Figure 11. The first (0, 1) mode shapes and vibrational frequency response characteristics of ultrathin wood film under sound wave excitations of different directions.

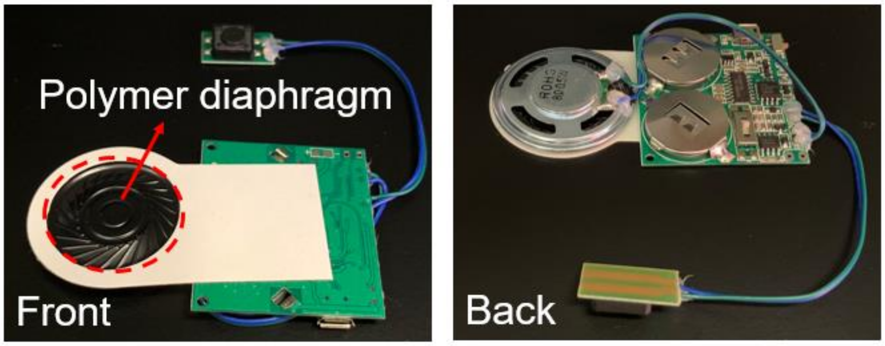

Supplementary Figure 12. The commercial speaker purchased from Shenzhen Maiyout Technologu Co., Ltd (MY-H1650).

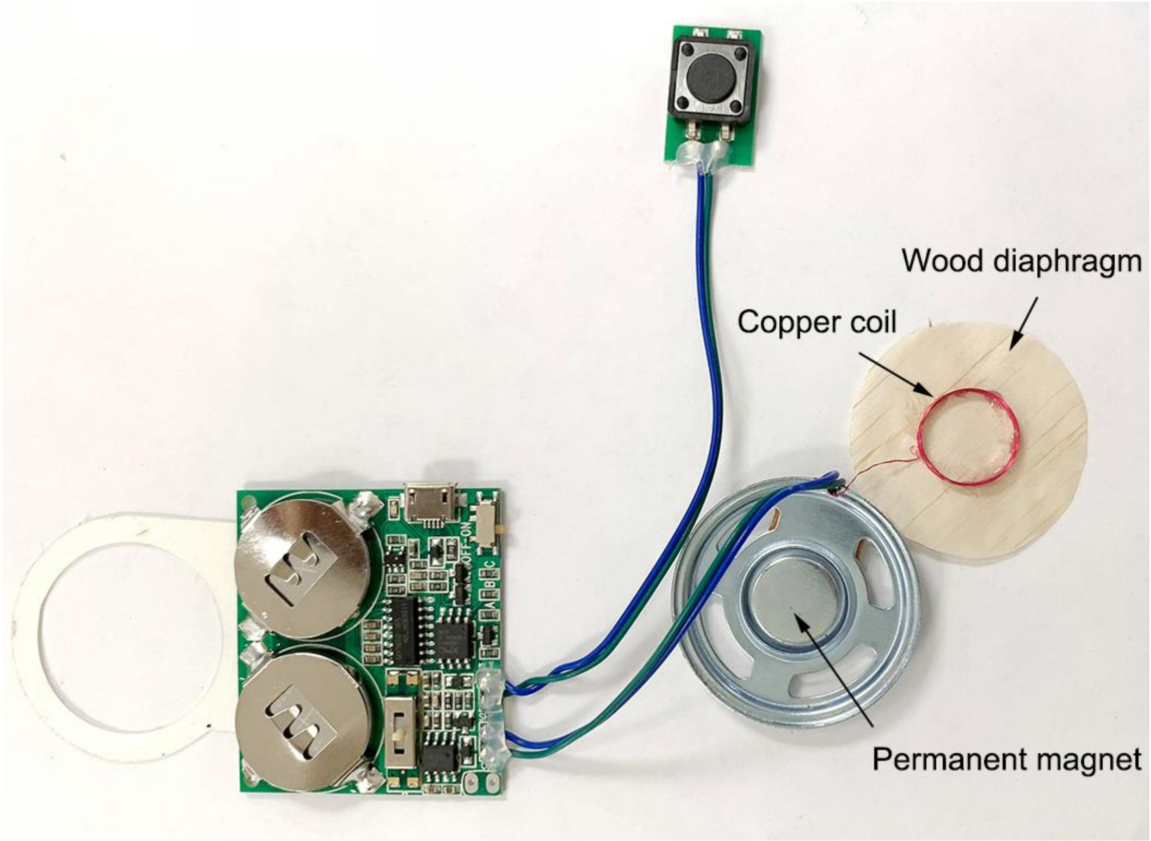

Supplementary Figure 13. Photograph of the speaker prototype made with the ultrathin wood film diaphragm. The electromagnetic forces act on the coil, allowing it to vibrate the diaphragm back and forth, in this manner translating the electrical signal into an audible sound.
